# Bioethanol Production from UK Seaweeds: Investigating Variable Pre-treatment and Enzyme Hydrolysis Parameters

**DOI:** 10.1007/s12155-019-10054-1

**Published:** 2019-10-26

**Authors:** Emily T. Kostas, Daniel A. White, David J. Cook

**Affiliations:** 1 International Centre for Brewing Science, Division of Food Science, The University of Nottingham, Sutton Bonington Campus, Loughborough, Leicestershire, LE12 5RD UK; 2 Department of Biochemical Engineering, The Advanced Centre of Biochemical Engineering, Bernard Katz Building, University College London, Gower Street, London, WC1H 6BT UK; 3 Plymouth Marine Laboratory, Prospect Pl, Plymouth, Devon PL1 3DH UK

**Keywords:** Macroalgae, Pre-treatment, Bioethanol, *L. digitata*, *D. carnosa*, *U. lactuca*

## Abstract

This study describes the method development for bioethanol production from three species of seaweed. *Laminaria digitata*, *Ulva lactuca* and for the first time *Dilsea carnosa* were used as representatives of brown, green and red species of seaweed, respectively. Acid thermo-chemical and entirely aqueous (water) based pre-treatments were evaluated, using a range of sulphuric acid concentrations (0.125–2.5 M) and solids loading contents (5–25 % [w/v]; biomass: reactant) and different reaction times (5–30 min), with the aim of maximising the release of glucose following enzyme hydrolysis. A pre-treatment step for each of the three seaweeds was required and pre-treatment conditions were found to be specific to each seaweed species. *Dilsea carnosa* and *U. lactuca* were more suited with an aqueous (water-based) pre-treatment (yielding 125.0 and 360.0 mg of glucose/g of pre-treated seaweed, respectively), yet interestingly non pre-treated *D. carnosa* yielded 106.4 g g^−1^ glucose. *Laminaria digitata* required a dilute acid thermo-chemical pre-treatment in order to liberate maximal glucose yields (218.9 mg glucose/g pre-treated seaweed). Fermentations with *S. cerevisiae* NCYC2592 of the generated hydrolysates gave ethanol yields of 5.4 g L^−1^, 7.8 g L^−1^ and 3.2 g L^−1^ from *D. carnosa*, *U. lactuca* and *L. digitata*, respectively. This study highlighted that entirely aqueous based pre-treatments are effective for seaweed biomass, yet bioethanol production alone may not make such bio-processes economically viable at large scale.

## Introduction

The United Kingdom can be regarded as a centre for seaweed diversity in the North Atlantic where over 650 different species of red, green and brown seaweed inhabit the British coastline [1]. This represents ca. 50% and ca. 7% of both the north Atlantic and globally documented species, respectively [1]. However, despite this high abundance of inherent species of seaweeds and the potential for sustainable cultivating and harvesting, British seaweeds are an underutilised resource that could potentially be incorporated into bio-processes. These bio-processes can include the production of bioethanol and the development of seaweed biomass as a feedstock for biofuel production is currently a global area of research. Efforts are being made to develop technological innovations that can assist in both improving seaweed cultivation and improving biomass conversion process efficiencies in order to generate bioethanol from seaweed feedstocks on a commercial scale [2].

Establishing a strategy that will successfully and efficiently achieve the complete hydrolysis of seaweed polysaccharides (liberating fermentable sugars) is a complex task and although bioethanol can be produced from seaweed, it has been challenging to obtain sufficiently high concentrations of ethanol [3, 4]. This is mainly because the biochemical structure and composition of seaweeds differ greatly to land-based plants, with variability existing between seaweed species belonging to different taxonomical groups (such as the diverse array of polysaccharides). As such, obtaining adequate quantities of fermentable sugars has been regarded as one of the main bottlenecks. Furthermore, the technologies developed for lignocellulosic plants are not compatible with seaweed feedstocks [5] and cannot be applied. For example, seaweeds contain proportions of starch and cellulose (as found in terrestrial plants), however, the majority of the inherent carbohydrates are in the form of alternative polysaccharides such as laminarin and alginate (in brown spp.), carrageenan and agarose (in red spp.) and ulvan (in green spp.) [6].

Bioethanol production from seaweed resources typically follow conventional methods of biomass hydrolysis which were primarily developed specifically for use with terrestrial plants for both first- and second-generation bioethanol production [7]. Conventional methods usually include the use of a single stage chemical based hydrolytic pre-treatment of the biomass, by employing either acid, alkali and even organic solvents as catalysts to directly release fermentable sugars which can then be fermented to bioethanol [8–11]. The uses of a range of acids and concentrations have been extensively explored to optimise the hydrolysis of seaweed polysaccharides [12–14]. This has often been investigated with different combinations of reaction times, temperatures and pressures as these parameters have shown to influence the maximum final yields of sugar that are liberated from the seaweed biomass [12, 15, 16]. Studies have also proved that a two stage process (an initial acid chemical pre-treatment followed by subsequent enzyme hydrolysis; similar to that commonly conducted for lignocellulosic biofuels) can increase the yields of fermentable sugars by at least double [17, 18]. For example, Ra et al. [19] added a blend of enzymes (Viscozyme-L and Celluclast; Novozymes, Denmark) for the hydrolysis of a pre-treated slurry of red algal species *G. verrucosa*, liberating 84.2% of the theoretical maximum of the species’ total carbohydrate content [19]. Li et al [5] applied a hydrogen peroxide pre-treatment on *Ulva prolifera* biomass which ultimately improved the efficiency of enzyme hydrolysis on the residue, achieving a maximum reducing sugar yield of 0.42 g g^−1^ residue under optimal pre-treatment conditions [5]. Although acid-catalysed thermal pre-treatment is commonly used on seaweeds and is favourably cost effective, its application does have potential disadvantages such as high energy consumption and also the potential for environmental pollution. Furthermore, such chemical pre-treatments that are coupled with higher temperatures and/or longer reaction times (to maximise the efficiency of this hydrolytic step) may also often lead to the formation of degradation products. The accumulation of sugar-derived by-products such as 5-hydroxymethylfurfural (HMF) and furfural, and the generation of organic acids (such as formic and levulinic acids) may have inhibitory effects on downstream processes, such as the enzymatic reactions and fermentations [20]. Although the presence of the aforementioned degradation products have been quantified in lower levels in seaweed hydrolysates [6]. Current research is seeking to establish new, eco-friendly ‘green’ technologies that can overcome the disadvantages of thermo-chemical pre-treatments (high energy input, increased risk of thermal degradation products, suitable equipment), and a potential alternative could be the use of hydroxyl radicals. Auto-hydrolytical (water based) pre-treatments could be a plausible variant as the reaction is catalysed by the hydronium ion and subsequent organic acids which are consequently generated (such as acetic acid) [21]; without the requirement of any additional reagents. Despite the environmentally friendly nature of auto-hydrolytical pre-treatments, only a handful of studies have employed this form of pre-treatment to seaweed, with both studies focussing on brown species *Macrocystsis pyrifera* [22] and *Sargassum muticum* [23] for the production of bioethanol. In order to efficiently produce bioethanol from any species of seaweed, particularly for commercial scale, it is imperative to apply a cost-effective and sustainable, but importantly, compatible pre-treatment in order to enhance the liberation of fermentable sugars. However, due to the differences in biochemical composition and structural arrangements that exist between both inter-species and taxonomically, the same pre-treatment, in theory, cannot be applied. Therefore further research is needed to fine-tune and optimise pre-treatment protocols for each seaweed species.

**Fig. 1:**
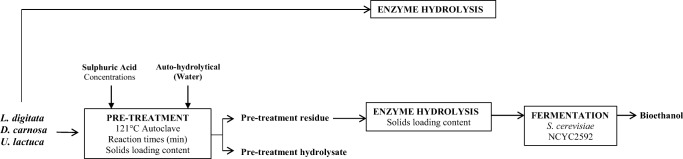
Schematic flow diagram used in this study

This research describes the method development of bioethanol production methodologies for seaweeds belonging to three different taxonomical groups; *Laminaria digitata*, *Dilsea carnosa* and *Ulva lactuca*. The bioethanol production systems explored here utilised an initial pre-treatment, then an enzymatic hydrolysis step for maximising glucose liberation, followed by fermentation (following the scheme displayed in Fig 1). In addition to using water as the pre-treatment reagent, a range of sulphuric acid concentrations (0.125–2.5 M) were evaluated at 121 °C for varying reaction times (5–30 min). Direct enzyme hydrolysis alone and combinations of either acid (or water) and enzyme hydrolysis were also examined in order to identify the most suitable pre-treatment for each of the three seaweeds, as well as an initial attempt at enhancing the enzyme hydrolysis step. Not only did this study aim to identify the most suitable pre-treatment conditions and/or combinations for each species of seaweed investigated, it also sought to highlight potential processing issues and discuss viability at larger scale.

## Materials and Methods

### Reagents

All reagents were of analytical grade and obtained from Sigma-Aldrich (UK) and Fisher Scientific (UK) unless otherwise specified. All water used was subjected to deionised reverse osmosis and of ≥ 18 mega-ohm purity.

### Seaweed Collection and Preparation

The seaweeds used in this study (*L. digitata*, *D. carnosa* and *U. lactuca*) were collected at spring low tides in May 2013 near Downderry in Cornwall (GPS coordinates 50.3620°N. 4.3667°W) in 2013. The seaweed samples were rinsed in distilled water to remove salt and debris, and then dried in a fan oven at 40 °C for a minimum of 48 h until dry. The seaweed samples were then milled using a ball mill (Fritsch, Germany) to obtain a fine homogeneous powder and stored in a desiccator away from direct sunlight and moisture until further analysis.

### Compositional Analysis of Seaweeds

Moisture content was measured by drying 5 g in a convection oven at 130 °C for 90 min [24]. After heating, the samples were placed in a desiccator for 30 min to prevent rehydration before being weighed again.

Ash content was determined by heating in a muffle furnace at 580 °C for 24 h.

A Thermo Flash Nitrogen Analyser (ThermoFischer Scientific, Waltham, Massachusetts, USA) was used to determine the protein content of each species. Sample (50 mg) was sealed in a tin capsule and combusted at approximately 1800 °C. Combustion gases were passed into a reduction reactor (at 680 °C and containing reduced copper) where nitrogen oxides were converted to elemental nitrogen. Carbon dioxide, sulphur dioxide and water were removed via filters of soda lime, magnesium perchlorate and a molecular sieve. The effluent stream was passed through a nitrogen separation column (50 °C) and into a thermal conductivity detector. Quantification was achieved with Eager 300 software using an l-aspartic acid standard. Protein was determined using the *N* × 6.25 conversion factor.

Lipid content was determined using an adaption of the Folch method [25]. Powdered sample (400 mg) was added to a 50-mL glass centrifuge tube to which 12 mL of dichloromethane/methanol (2:1, v/v) was added and left for 2 h with occasional agitations. The glass tubes were then centrifuged at 123 g for 5 min or until a pellet had formed at the bottom of the tube. Using a glass syringe, the upper organic phase was removed and transferred into a clean 50-mL centrifuge glass tube where 2.5 mL KCl (0.88 %, w/v) was added before being inverted, vortexed and centrifuged at 123 g for 5 mins. The lower organic phase was removed using a glass syringe and transferred into a pre-weighed glass tube. The lower organic phase was dried with nitrogen gas and left uncapped in a fume cupboard overnight until all the liquid had evaporated.

Total carbohydrates were measured following the assay outlined by Dubois et al (1956) where 1 mL of 12 M H_2_SO_4_ was added to 30 mg of seaweed in a heat-resistant screw cap glass tube and incubated at 37 °C for 1 h [26]. Water (11 mL) was added to the sample to dilute the acid strength to 1 M, following which, samples were incubated at 100 °C for 2 h. Liberated monosaccharides (mannitol, fucose, arabinose, galactose, glucose and xylose) were analysed by HPAEC-PAD as described in “Analysis of Monosaccharides, Inhibitors and Bioethanol”. Carbohydrate content was measured as the total of monosaccharides; glucose, galactose, mannitol, fucose, xylose and arabinose.

For multi element analysis, around 200 mg of each species of seaweed were weighed into digestion vessels to which 6 mL of HNO_3_ (concentrated) was added. The digestion vessels were then placed into a microwave rotor (Anton Paar Multiwave Pro 24HVT50) where they were heated to 140 °C for 20 min and then cooled at 55 °C for 15 min. Once the digestion was complete, water was added to make a final volume of 20 mL. Seaweed samples were then transferred to a universal storage bottle and stored at 4 °C until analysis. For the quantification of iodine, samples were prepared according to the method of Watts and Mitchell [27]. Samples (250 mg) were weighed into Pyrex tubes, to which 5 mL of 5 % (v/v) tetramethylammonium hydroxide (TMAH) was added. Samples were shaken before being placed into a convection oven at 70 °C for 3 h, with bottles shaken at 1.5 h. RO water (5 mL) was added to the samples after the 3-h incubation period, and the samples were transferred to 50-mL centrifuge tubes and centrifuged at 4696 g for 25 min. The supernatant was diluted to a final concentration of 1% (v/v). All analyses were conducted in triplicate. All trace multi-element analysis was performed on an ICP-MS (Thermo-Fisher Scientific iCAP-Q, Germany) equipped with a Flatopole collision cell upstream of the analytical quadrupole to reduce polyatomic interferences. Internal standards were introduced to the sample stream via a T-piece and typically included Sc (50 μg L^−1^), Ge (20 μg L^−1^), Rh (10 μg L^−1^) and Ir (5 μg L^−1^) in the preferred matrix of 2 % HNO_3_. External calibration standards were all in the range 0–100 μg L^−1^. Samples were introduced via a covered autosampler (Cetac ASX-520) through a concentric glass venturi nebuliser (Thermo-Fisher Scientific) or a PEEK Burgener Miramist nebuliser. Sample processing was undertaken using Qtegra software (Thermo-Fisher Scientific).

### Thermo-Chemical and Auto-hydrolytical Pre-treatment Investigation of Seaweed

#### Acid Thermo-chemical and Auto-hydrolytical (Water) Pre-treatment of Seaweed Feedstocks

Homogenised (powdered) samples (1.0 g) of *L. digitata*, *D. carnosa* and *U. lactuca* were each separately loaded into sealed glass Pyrex reaction tubes for heating in a benchtop autoclave (Priorclave, Tactrol 2; RSC/E, London, UK). Preliminary experimentation of chemical pre-treatment was explored by using a *D-optimal* algorithm (Design Expert v 7.0, Stat-Ease Inc., Minneapolis, USA) to choose the treatment combinations for acid concentrations and reaction times within set parameters (Table 1). A range of sulphuric acid concentrations (0.125–2.5 M) and water, with a range of different reaction times (5–30 min) were investigated. Ten millilitres of either acid (0.125–2.5 M) or RO water was added to achieve the desired solids loading of 10% (w/v). The reaction tubes were placed in the benchtop autoclave set to 121 °C and heated for 5–30 min. Experimental conditions were conducted in triplicate.

**Table 1:** Experimental design matrix for the investigation of the dilute acid hydrothermal pre-treatment of seaweed biomass in a bench-top autoclave

Experimental	Factor A	Factor B
Run order	H_2_SO_4_ acid (M)	Reaction time (min)
1	0.125	5
2	0.75	11
3	1.25	5
4	1.25	18
5	1.75	11
6	0.125	30
7	0.125	30
8	2.5	18
9	0.125	18
10	1.25	30
11	1.25	18
12	2.5	5
13	2.5	30
14	2.5	30
15	1.25	18
16	2.5	5
17	1.75	30
18	0.125	18
19	0.75	5
20	0.75	18
21	0.75	30
22	1.75	5
23	0.125	24
24	1.75	24
25	2.5	24
26	2.5	11
27	0.75	24
28	0.125	24

The reaction vessels were left to cool to ambient temperature before being centrifuged at 4696 g for 25 min in order to separate the solid seaweed material (referred to as pre-treated seaweed residues) from the acid and water generated hydrolysates. The hydrolysates were then passed through a 0.45 μm filter (Whatman Ltd, UK) and analysed for monosaccharide and degradation product liberation (“Analysis of Monosaccharides, Inhibitors and Bioethanol”) where amounts were expressed on a mg per g seaweed basis. The recovered pre-treated seaweed residues were exhaustively washed with 50 mL RO water (three rounds of centrifugation at 4696 g for 5 min then discarding the supernatant) to remove any degradation products that could have been generated during the pre-treatment step, and left to dry in a convection oven at 40 °C until dry. Dried pre-treated seaweed residues were compared to the quantity of seaweed initially loaded into the Pyrex reaction tube in order to determine mass loss. Dried samples were then stored in an air-tight environment away from direct sunlight until further analysis in “Determining the Efficacy of the Pre-treatment Conditions on the Seaweed Feedstocks”.

### Determining the Efficacy of the Pre-treatment Conditions on the Seaweed Feedstocks

Subsamples (0.1 g) of dried pre-treated seaweed residues (generated in Acid Thermo-Chemical and Auto-hudrolyical (Water) Pre-treatment of Seaweed Feedstocks) were mixed with 20 mL of 50 mM sodium citrate buffer (pH 5) and dosed with an excess (ca. 50 FPU/g biomass) of Novozymes Cellic® CTec2. The samples were then incubated at 50 °C for 48 h in a shaking incubator set at 120 rpm. Amounts of glucose present in the enzyme liquid fraction were quantified by HPAEC-PAD (“Analysis of Monosaccharides, Inhibitors and Bioethanol**”**) and calculated as the amount (mg) liberated from 1 g of dried pre-treated seaweed residue. Higher glucose yields obtained from the enzymatic saccharification were indicative of a more effective pre-treatment. The glucose concentration present in the Novozymes Cellic® CTec2 enzyme preparation was analysed and subtracted from each enzymatic sugar yield to allow for accurate calculation of yields. All experiments were conducted in triplicate.

### Pre-Treatment Investigation — Evaluating the Effect of Variable Solids Loading (Seaweed: Liquid)

Dried whole seaweed samples (0.5, 1.0, 1.5, 2.0 and 2.5 g; prepared as described in “Seaweed Collection and Preparation”) were accurately weighed into Pyrex reaction tubes and mixed with the optimal reaction catalyst (either H_2_SO_4_ or water) to give final solids loading rates of either 5, 10, 15, 20 and 25 % (w/v). Reactions were conducted in triplicate. The samples were then heated in a benchtop autoclave at 121 °C for the previously determined reaction times. After the samples were cooled to ambient room temperature, the pre-treatment generated hydrolysates were separated from the remaining seaweed residues by centrifugation as outlined in Acid Thermo-Chemical and Auto-hudrolyical (Water) Pre-treatment of Seaweed Feedstocks. Pre-treatment generated hydrolysates were analysed by HPAEC-PAD to determine the concentration of liberated monosaccharides from the seaweed biomass (“Analysis of Monosaccharides, Inhibitors and Bioethanol”). Pre-treated seaweed residues were then washed with RO water, dried and the weight recorded to determine mass loss. Enzymatic hydrolysis (using Novozymes Cellic® CTec2) of the pre-treated residues were then conducted to determine the glucose content that could be liberated from these pre-treated seaweed residues (as explained in “Determining the Efficacy of the Pre-treatment Conditions on the Seaweed Feedstocks”).

### Enzyme Hydrolysis — Evaluating the Effect of Variable Solids Loading (Seaweed Residue: Enzyme Buffer)

Dried pre-treated seaweed samples (0.4, 0.8, 1.6 and 3.2 g; obtained from Pre-treatment Investigation - Evaluating the Effect ofVariable Solids Loading (Seaweed: Liquid)) were accurately weighed into 50-mL centrifuge tubes and mixed with 50 mM sodium citrate buffer (pH 5) to give final solid loading rates of 2, 4, 8 and 16 % (w/v) (experimental volume of 10 mL). The same amount of excess (ca. 50 FPU/g biomass) of Novozymes Cellic® CTec2 was dosed into each vessel and gently inverted before being incubated at 50 °C for 48 h at 120 rpm. All experiments were conducted in triplicate. Enzyme liquid fraction were separated from the seaweed residue by centrifugation at 4696 g for 25 min and then filtered through a 0.45 μm filter (Whatman Ltd, UK). The liquid fraction was run on HPAEC-PAD (“Analysis of Monosaccharides, Inhibitors and Bioethanol”) to determine liberated glucose yields.

### Fermentation of Seaweed Enzymatic Hydrolysates

Fermentations of the hydrolysate generated after applying the most suitable pre-treatment and enzyme hydrolysis conditions for each species of seaweed (25 mL total volume) were conducted in glass serum bottles (30 mL capacity; Wheaton, USA) using a method adapted from Quain et al. [28] and Powell et al. [29]. The vessels were made anaerobic by sealing the vessels with rubber septa. A one way valve was used in order to facilitate the expulsion of any CO_2_ produced during the fermentation process as sugars were converted to ethanol. The fermentation vessels were inoculated with *S. cerevisiae* strain NCYC 2592 at a pitching rate of ca 10^7^ cell mL^−1^. The fermentation vessels were then incubated at 30 °C (MIR-253 incubator, Sanyo Electric Co., Japan) with magnetic stirring (Cimarec™ Multipoint Stirrer, Thermo Scientific™, UK) set at 120 rpm and the progression of the fermentation was monitored by tracking the weight loss (resulting from the removal of CO_2_) of the vessels at frequent intervals. The end of the fermentation (attenuation) was indicated by the vessels reaching constant mass. Samples were taken at the end of the fermentation for glucose and ethanol quantification via HPLC (“Analysis of Monosaccharides, Inhibitors and Bioethanol”). All fermentations were carried out in triplicate.

### Analysis of Monosaccharides, Inhibitors and Bioethanol

The monosaccharide concentrations were quantified via HPAEC-PAD using Dionex ICS-3000 Reagent-Free™ Ion Chromatography, electrochemical detection using ED 40. The CarboPacTM PA 20 column (3 × 150 mm, Dionex, USA) was used and the mobile phase was 10 mM NaOH with a flowrate of 0.5 mL min^−1^. The injection volume was 10 μL and the column temperature was 30 °C. Authentic standards of monosaccharides (mannitol, fucose, arabinose, galactose, glucose and xylose) over a concentration of 1 to 0.0625 g L^−1^ were used for reference and quantification.

The analysis of inhibitors (HMF, furoic acid, furfural, vanillic acid, vanillin, ferulic acid and *p*-coumaric acid) were quantified by HPLC using UV detection at 270 nm (2695 HPLC system and 996 Photodiode Array Detector, Waters, USA) and a Techsphere ODS C18 column (5 μm, 4.6 mm × 250 mm; HPLC Technologies, UK) was used at room temperature. The sample volume was 10 μL and the mobile phase was a gradient of methanol in 1 % acetic acid at an overall flow rate of 1.0 mL min^−1^. The methanol concentration increased linearly from 20 to 50 % over 30 min with a 100 % methanol column cleaning phase and a 9 min re-equilibration period. Data were recorded using Millennium Chromatography software (Waters, USA).

Ethanol yields produced during fermentation were quantified by HPLC using an AS-2055 Intelligent Auto-sampler and a PU-1580 Intelligent HPLC Pump (Jasco, Japan). The Rezex ROA Organic Acid H+ organic acid column (5 μm, 7.8 mm × 300 mm; Phenomenex, UK) was operated at ambient temperature with a mobile phase of 0.0025 M H_2_SO_4_ at a flow rate of 0.5 mL min^−1^. A Refractive Index cell (RI-2031 Intelligent Refractive Index detector, Jasco, Japan) was used for detection and the injection volume was 10 μL. Data were acquired using the Azur software package v. 4.6.0.0 (Datalys, France). Prior to HPLC analysis, all samples and standards were filtered using Whatman GD/X syringe filters (GF/C 25 mm filter diameter/1.2 μm pore size; Whatman International Ltd., Banbury, UK).

## Results and Discussion

### Composition of Seaweed Species

**Fig. 2:**
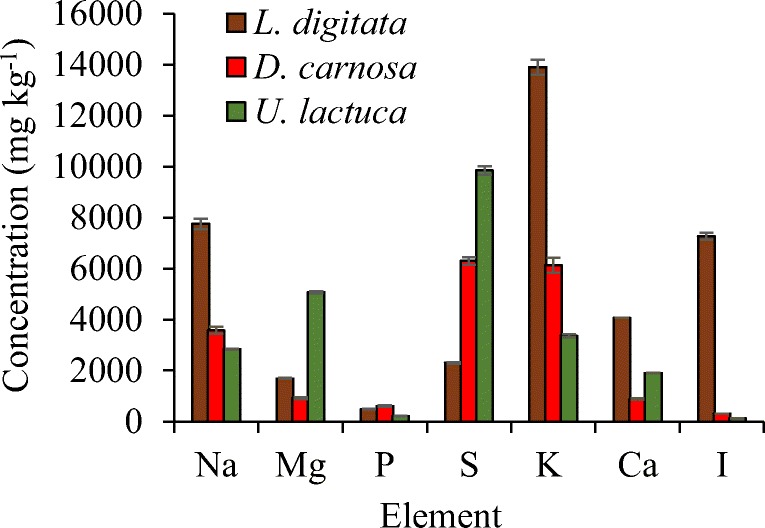
Liberation of glucose from pre-treated. **A**) *L. digitata*. **B**) *D. carnosa*. **C**) *U. lactuca* residues. Enzymatic hydrolysis conducted using Novozymes Cellic® CTec2 dosed at 50 FPU/g biomass at 50 °C for 48 h with a solids loading of 0.5% (w/v). Data are the mean±SD of three measurements

The compositions of the three seaweed species investigated in this study are listed in Table 2. The ash content was relatively high, ranging from 15.4 to 24.3% dry weight (d/w). The protein content was within the range of 16.4–26.8% (d/w) and the lipid content within values ranging from 1.0 to 1.9% (d/w) across the three species. *Dilsea carnosa* had the highest carbohydrate content of 41.8%, whereas *U. lactuca* contained 23.8 % carbohydrate. *Laminaria digitata* had the lowest carbohydrate content of 21.7% ± 0.68 (d/w) which may be due to the fact that the seaweeds were harvested in May for this study. It has been well documented that the composition of seaweed carbohydrates is variable and dependent on when in the season they were harvested [30–32]. Adams et al. [33] revealed that the most suitable month to harvest *L. digitata* from UK waters for bioethanol production was July, as this is when carbohydrate yields (mainly mannitol and laminarin) were found to be highest. This could ultimately influence the final yields of bioethanol that can be achieved and also represents the possibility for further improvements. Furthermore, it is also possible that some monosaccharides (e.g. mannuronic and guluronic acids that originate from the polysaccharide alginate) were released (during the carbohydrate quantification assay) but were not quantified using the analytical method applied. The concentrations of the main identified elements are shown in Fig. 2 and it is evident that levels varied across the three seaweeds. *Laminaria digitata* had the highest levels of sodium, potassium and iodine, whereas *U. lactuca* contained greater levels of magnesium and sulphur. *Dilsea carnosa* had the lowest levels of all identified elements.

**Table 2:** Biochemical composition of seaweed species used in this study

Seaweed species	Ash	Composition % (dry weight basis)			
		Protein	Lipid	Carbohydrate^a^	Moisture
*L. digitata*	24.3 ± 0.38	26.8 ± 0.19	1.9 ± 0.09	21.7 ± 0.68	12.1 ± 0.39
*D. carnosa*	15.4 ± 025	22.2 ± 0.64	1.3 ± 0.70	41.8 ± 0.64	0.8 ± 0.49
*U. lactuca*	21.5 ± 0.29	16.4 ± 0.14	1.0 ± 0.23	23.8 ± 0.80	10.0 ± 0.01

^a^Carbohydrate was estimated as the sum of monosaccharides arabinose, galactose, glucose, xylose, fucose and mannitol. It is assumed that the unaccounted for dry matter is principally polysaccharide material either not broken down under the hydrolysis conditions employed or not quantified against authentic standards during HPAEC-PAD analysis. Data are the mean ± SD of three measurements

### Investigation of Pre-treatment Conditions

#### Identification of Suitable Pre-treatment Catalyst and Conditions for Each Species of Seaweed

Six representative sample preparations from the experimental design mix as detailed in Table 1 were selected for the quantification of glucose in the remaining solid residues generated after thermal pre-treatment. Suitable thermal pre-treatment conditions were identified based on the maximum liberation of glucose from the treated residue after subsequent enzymatic hydrolysis with an excess of Novozymes Cellic® CTec2. This enzyme cocktail was chosen due to the unavailability of a more ‘seaweed-specialised’ enzyme blend. Although the seaweeds investigated in this study contain hexose (C6) monosaccharides other than glucose (such as galactose) that are also readily fermented by yeast, it was decided to focus solely on the liberation of glucose as a performance metric. This was due to the fact that glucose is the primary hydrolysis product which is released using this proprietary enzyme blend (which predominately contains cellulases). Whilst this did not reflect a commercially viable process it provided a best case scenario for achieving maximal glucose yields and was used purely for proof of principal.

**Fig. 3:**
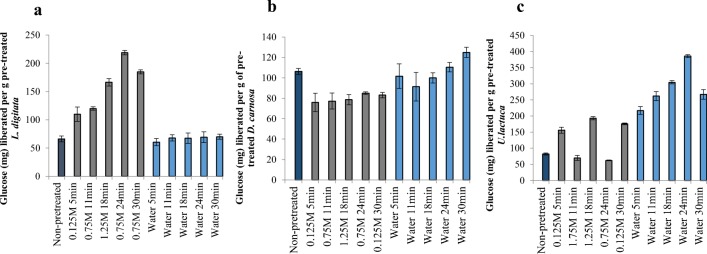
Elemental analysis of *L. digitata*, *D. carnosa* and *U. lactuca*

Sulphuric acid-based pre-treatments liberated c.a. 110.0–218.9 mg of glucose per gram of pre-treated *L. digitata* (Fig. 3A) after enzymatic hydrolysis. The highest amount of glucose (218.9 mg g^−1^) was liberated from a residue that was produced after treatment with 0.75 M sulphuric acid for 24 min at 121 °C. From this pre-treatment, HMF was the only detected degradation product at a concentration of 3.0 mg per gram of *L. digitata* found in the pre-treatment hydrolysate fraction (Table 3). In contrast, auto-hydrolytical (aqueous only) based pre-treatments liberated lower glucose concentrations (60.5–70.1 mg g^−1^); however, no degradation products were identified in the generated pre-treatment hydrolysates. Mass losses for acid pre-treated *L. digitata* ranged from 54.0–70.8%, whereas for the auto-hydrolytical treatments the mass losses were lower (52.0–62.9%). Furthermore, there also appeared to be no difference in the release of glucose between the non-pre-treated and auto-hydrolytically pre-treated *L. digitata* residues. This suggests that auto-hydrolytical pre-treatment on *L. digitata* does not structurally modify the surface of the seaweed (for easier Novozymes Cellic® CTec2 access) at all; validating the requirement of acid for pre-treatment. SEM analysis of both non-pre-treated *L. digitata* and auto-hydrolytically pre-treated residues of *L. digitata* would confirm this.

**Table 3:** Levels of degradation products (HMF and furfural) in the pre-treatment hydrolysates and mass losses of *L. digitata*, *D. carnosa* and *U. lactuca*

Pre-treatment conditions at 121 °C	*L. digitata*		*D. carnosa*			*U. lactuca*		
	HMF (mg g^−1^)	Mass loss (%)	HMF (mg g^−1^)	Furfural (mg g^−1^)	Mass loss (%)	HMF (mg g^−1^)	Furfural (mg g^−1^)	Mass loss (%)
0.125 M 5 min	n.a	54.2 ± 1.02	n.a	n.a	75.0 ± 4.5	n.a	n.a	71.3 ± 0.6
0.75 M 11 min	0.4 ± 0.1	67.6 ± 0.3	n.a	n.a	77.9 ± 6.1	n.a	n.a	77.3 ± 0.6
1.75 M 11 min	n.a	76.3 ± 1.2	n.a	n.a	80.8 ± 0.6	0.2 ± 0.025	0.2 ± 0.04	76.6 ± 1.2
1.25 M 18 min	5.0 ± 1.0	74.6 ± 3.0	0.1 ± 0.02	0.2 ± 0.005	81.6 ± 4.3	0.4 ± 0.06	0.3 ± 0.02	79.2 ± 1.1
0.75 M 24 min	3.0 ± 1.0	70.8 ± 1.6	0.1 ± 0.01	0.2 ± 0.009	79.4 ± 2.5	0.4 ± 0.009	0.2 ± 0.01	77.9 ± 0.9
0.75 M 30 min	3.0 ± 0.5	70.8 ± 2.3	n.a	n.a	88.5 ± 1.7	n.a	n.a	85.2 ± 1.6
Auto-hydrolytical 5 min	0.0	52.1 ± 0.9	0.0	0.0	58.1 ± 1.9	0.0	0.0	59.6 ± 2.6
Auto-hydrolytical 11 min	0.0	57.3 ± 3.1	0.0	0.0	61.2 ± 6.1	0.0	0.0	59.9 ± 1.4
Auto-hydrolytical 18 min	0.0	59.6 ± 2.0	0.0	0.0	60.1 ± 0.6	0.0	0.0	60.8 ± 0.3
Auto-hydrolytical 24 min	0.0	60.6 ± 1.7	0.0	0.0	60.3 ± 1.2	0.0	0.0	61.7 ± 1.8
Auto-hydrolytical 30 min	0.0	62.9 ± 2.1	0.0	0.0	63.1 ± 1.6	0.0	0.0	65.4 ± 2.6

mg g^−1^; mg of degradation product per g of treated seaweed

Data are the mean ± SD of three measurements

On the contrary, auto-hydrolytical based pre-treatments slightly improved glucose liberation yields for *D. carnosa* compared to the sulphuric acid based pre-treatments (Fig. 3B), with an auto-hydrolytical treatment of 30 min at 121 °C yielding the greatest amount of glucose (125.0 mg g^−1^). Autohydrolytical pre-treatment mass losses of *D. carnosa* were within the range of 58.1–63.1%. A significant amount of research has been conducted on bioethanol production from red seaweeds, of which sulphuric acid has been identified to be the most suitable reaction catalyst for the initial pre-treatment stage. This includes studies performed on *Kappaphycus alvarezii* [15, 34] *Gelidium amansii* [35–37], *Gracilaria verrucosa* [38], *Gracilaria tenuistipitata* [36], *Gelidium elegans* [39] and *Gracilariopsis chorda* [36], where sequential two stage pre-treatments (acid pre-treatment followed by enzyme hydrolysis) have been applied, subsequently followed by fermentation to bioethanol. A study by Nguyen et al [35] who identified optimal conditions (180 mM sulphuric acid at 121 °C for 45 min at a solids loading content of 12% w/v) for liberating glucose (6.8 g L^−1^) and galactose (26.1 g L^−1^) from *G. amansii* included an additional detoxification step in order to remove HMF which had reached levels as high as 8.7 g L^−1^ as a result of the thermal acid pre-treatment. Although HMF and furfural were detected in this study, they were present at extremely low concentrations around 0.1 and 0.2 mg g^−1^, respectively and only in the pre-treatment hydrolysates generated from sulphuric acid (Table 3), making auto-hydrolytical pre-treatment an attractive alternative to thermal acid pre-treatments. Interestingly, approximately 106.4 mg g^−1^ of glucose was directly released from non-pre-treated (native) *D. carnosa* after direct hydrolysis with Novozymes Cellic® CTec2; calling into question the actual requirement for any form of pre-treatment. It is not clear why this may be and no published literature on bioethanol production from *D. carnosa* were currently available. The authors believe that *D. carnosa* may consist of structural complexes (in particular the specific ultrastructure of cellulose present) which could be less recalcitrant than the other two seaweeds. As such, milder pre-treatments (if any at all) may be better suited to this species and bio-processing methodologies (bioethanol production processes) which have been optimised for other species of red seaweed cannot be directly applied to *D. carnosa* and function optimally.

Auto-hydrolytical pre-treatments appeared to outperform sulphuric acid pre-treatments for the green seaweed *U. lactuca* (Fig. 3C), additionally without the formation of degradation products in the pre-treatment hydrolysates. It may be possible that acid pre-treatment on *U. lactuca* was too harsh and could have altered the structure (mainly the cellulose fraction in the seaweed) in such a way making it non-compatible for the Novozymes Cellic® CTec2 blend to adequately function. Furthermore, glucose monomers may have been liberated from *U. lactuca* (hence the lower yields of glucose released from *U. lactuca* pre-treated residues after enzyme hydrolysis), however, could have subsequently been degraded to alternative sugar degradation products (such as levulinic acid) which were not detectable on the analytical system used in this work. Similarly to *D. carnosa*, auto-hydrolytical mass losses were between 59.6 and 65.4%. A pre-treatment for 24 min at 121 °C solubilised maximal glucose yields of 386.0 mg g^−1^ from the pre-treated *U. lactuca* residue.

Overall, it appeared that *U. lactuca* liberated the greatest yields of glucose (almost up to 400 mg g^−1^), followed by *L. digitata* (ca. 220 mg g^−1^) and lastly *D. carnosa* (125.0 mg g^−1^)*.* This is in agreement with the natural content of glucose containing polysaccharides that are typically associated with species of seaweed belonging to the three different taxonomical groups. Green species of seaweed, which are most closely related to land-plants, contain higher proportions of cellulose and hemicellulose, whilst brown species contain cellulose and β-1,3-glucan. Red seaweeds on the contrary contain smaller amounts of cellulose (and are mainly comprised of unique inherent polysaccharides, namely carrageenan and agarose) [3]. The ideal thermal pre-treatment conditions (for maximal glucose liberation) identified for each of the three species of seaweed and subsequently taken forward in this study were:

*L. digitata* – 0.75 M H_2_SO_2_, 24 min, 121 °C

*D. carnosa* – Auto-hydrolytical, 30 min, 121 °C

*U. lactuca* – Auto-hydrolytical, 24 min, 121 °C

#### Varying the Solids Loading Content (Seaweed: Reactant) During the Pre-treatment Stage

**Fig. 4:**
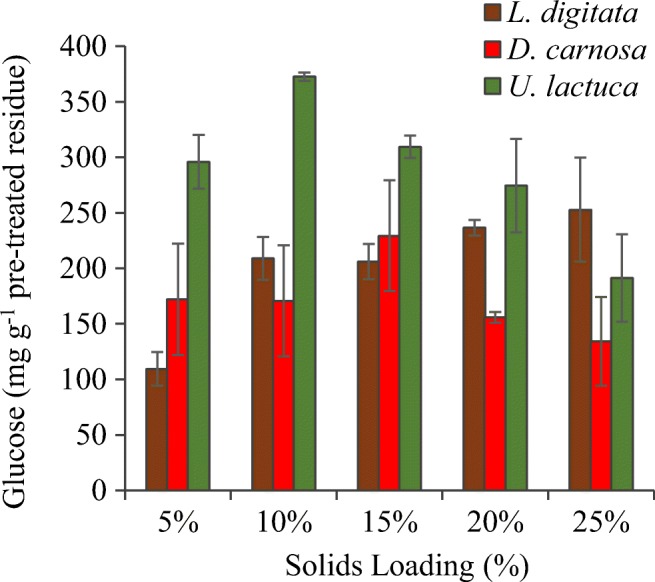
Effect of solids loading variation during pre-treatment of the three species of seaweed on glucose liberation (after enzyme hydrolysis). Pre-treatment: *L. digitata* (121 °C, 1.5 N H_2_SO_4_, 24 min). *D. carnosa* and *U. lactuca* (121 °C, auto-hydrolytical, 30 min and 24 min, respectively). Enzymatic hydrolysis conducted using Novozymes Cellic® CTec2 dosed at 50 FPU/g biomass at 50 °C for 48 h with a solids loading of 0.5% (w/v). Data are the mean±SD of three replicate measurements

The next set of experiments investigated the effects of variation of the solids loading content (biomass: reactant [w/v]) during the pre-treatment stage. Thermal pre-treatment conditions (reactant and time) that were most suitable for each seaweed from section “Identification of Suitable Pre-treatment Catalyst and Conditions for Each Species of Seaweed” were applied. Liberation of monosaccharides from the seaweeds into the pre-treatment hydrolysate can be seen in Table 4. Enzyme hydrolysis was also subsequently performed on the pre-treated seaweed residues for glucose liberation in order to measure pre-treatment efficacy and to help determine which solids loading content was most suitable for each species of seaweed (Fig. 4).

**Table 4:** Effect of solids loading variation on the liberation of monosaccharides directly into the pre-treatment hydrolysate

(mg g^−1^)^a^	*L. digitata*					*D. carnosa*^b^		*U. lactuca*				
	5%	10%	15%	20%	25%	5%	10%	5%	10%	15%	20%	25%
Mannitol	21.6 ± 3.2	17.6 ± 0.4	4.4 ± 0.1	2.6 ± 0.2	1.8 ± 0.1	< 1.0	< 1.0	< 1.0	< 1.0	< 1.0	< 1.0	< 1.0
Fucose	12.8 ± 0.1	8.8 ± 1.1	5.9 ± 0.7	5.8 ± 0.3	4.5 ± 0.4	0.0	0.0	0.0	0.0	0.0	0.0	0.0
Arabinose	< 1.0	< 1.0	< 1.0	< 1.0	< 1.0	0.0	0.0	0.0	0.0	0.0	0.0	0.0
Galactose	4.3 ± 0.4	2.5 ± 0.3	1.6 ± 0.1	1.4 ± 0.1	1.0 ± 0.1	0.0	0.0	0.0	0.0	0.0	0.0	0.0
Glucose	3.0 ± 0.1	1.7 ± 0.2	1.0 ± 0.1	< 1.0	< 1.0	0.0	0.0	< 1.0	< 1.0	< 1.0	< 1.0	< 1.0
Xylose	4.3 ± 0.3	3.1 ± 0.4	2.0 ± 0.2	1.7 ± 0.1	1.3 ± 0.1	0.0	0.0	< 1.0	< 1.0	< 1.0	< 1.0	< 1.0

^a^mg of monosaccharide released per g of seaweed directly into the pre-treatment hydrolysate

^b^No pre-treatment hydrolysates were generated from 15, 20 and 25 % solids loading tests, therefore no analysis was performed

The overall concentrations of monosaccharides in the post thermal pre-treatment hydrolysate decreased as the solids loading content increased beyond 5% (w/v); yet the liquid fractions still contained a similar profile of the different monomeric sugars (Table 4). Efficient heat transfer could be limited when operating at higher solids loadings rates and this is supported by the observation of decreased levels of mannitol in the acid pre-treatment hydrolysate fraction. Mannitol, which is highly soluble in water and is usually present at quite high levels in the hydrolysates generated after pre-treatment of seaweed [6, 40], can be easily extracted from brown seaweeds as it is found as a terminating side chain on the laminarin polymer [41]. Additionally, mannitol is easily extractable (together with fucoidan) from the cell wall (even without the need of physical cell disruption (lysis) and/or cell walls) of brown seaweed using hot water [42, 43]. However, as the *L. digitata* in this study had been washed, oven dried and milled to a powder before the pre-treatment tests were conducted, the cell walls would have been disrupted which would have aided the release of mannitol.

Around 252.9 mg glucose was liberated per g of pre-treated *L. digitata* (post enzyme hydrolysis) from a thermal pre-treatment that operated at a 25% solids loading content (Fig. 4). Processing at this high loading rate is advantageous as more *L. digitata* could be processed with any given thermal pre-treatment batch reaction and with less volume of acid reactant. Work performed by Sharma and Horn [44] also saw a similar trend when enzymatically hydrolysing *Saccharina latissima* at a 25 % solids loading rate, both with Novozymes Cellic® CTec2 alone and an enzyme blend composed of 90% Novozymes Cellic® CTec2 and 10 % Alginate lyase enzyme. The blend of the two enzyme liberated around 260 mg of soluble sugars per g of seaweed, whilst Novozymes Cellic® CTec2 hydrolysis alone yielded around 240 mg soluble sugars per gram. An interesting finding from their work was the identification of the effect of (seaweed) drying temperature on enzyme hydrolysis yields, with 30 °C appearing to be the most suitable temperature to dry the seaweed prior to enzyme hydrolysis. Furthermore, it has also been reported that combining Novozymes Cellic® CTec2 with commercial alginate lyase is not only effective at liberating all available glucose within 8 h [45] but it also decreases the overall viscosity within the first 1–2 h of the reaction [46] on *L. digitata* seaweed; this is advantageous for downstream processing of the hydrolysates containing glucose. Another study conducted on brown *Sargassum* spp suggested the optimum pre-treatment for this brown seaweed to be between 3.4–4.6% (w/v) sulphuric acid at 115 °C for 1.5 h and at 10% solids loadings [47]. However, enzyme hydrolysis with two different commercial enzyme mixtures (cellulase from *Trichoderma reeseii* ATCC 26921 and cellobiase (β-glucosidase) from *Aspergillus niger* Novozyme 188) only liberated ca. 45 mg g^−1^ glucose from the pre-treated seaweed biomass. Borines et al [47] included a washing step between thermal pre-treatment and enzyme hydrolysis which used hot water, instead of the ambient water used in this work. The use of hot water may have washed away easily soluble monosaccharides, especially glucose, ultimately reducing their final achieved yields of glucose.

Due to the hygroscopic nature of *D. carnosa* biomass (resulting from the presence of polysaccharides carrageenan and agar which have strong water gelling properties [48]), there was no recovery of an aqueous hydrolysate when conducting pre-treatments at high solids loading rates (15, 20 and 25 % (w/v)). Furthermore, the low volumes of pre-treatment hydrolysate that were recovered from the 5% and 10% solids loading reactions contained negligible amounts (< 0.1 mg g^−1^) of monomeric sugars (Table 4). A pre-treated residue of *D. carnosa* that underwent auto-hydrolytical treatment at 121 °C for 30 min with a 15% (w/v) solids loading liberated ca 229.4 mg glucose/g pre-treated residue post enzymatic hydrolysis (Fig. 4); the highest yield which appeared to be the optimum loading content for this species of seaweed. The effectiveness of the pre-treatment for *D. carnosa* diminished when solids loading increased above 15% (w/v). This may be due to the lower volume of water present which inevitably could have prevented adequate heating of the seaweed as the reaction was conducted without agitation.

For *U. lactuca,* it appeared that 10% solids loading was optimal as glucose yields of 372.6 mg g^−1^ were liberated post enzyme hydrolysis (Fig. 4). The use of an entirely aqueous (water) based hydrolysis method for the thermal pre-treatment of green seaweeds has not been previously reported [49–51]. Trivedi et al. [52] found that sodium acetate buffer alone (pH 4.8 at 120 °C for 60 min with a solids loading content of 5% (w/v)) enhanced the enzymatic hydrolysis (glucose) yields from the green seaweed *Ulva fasciata*. This form of mild acid pre-treatment yielded around 206 mg glucose per gram of the green seaweed; suggesting that the sodium acetate could have served as a catalyst for the deconstruction of the seaweed cell wall, or at least improved enzymatic access to the substrate. However, no entirely aqueous controls were run alongside to conclude whether it was the sodium acetate or the water that was responsible for the pre-treatment effect.

### Investigating the Solids Loading Ratio During the Enzymatic Hydrolysis of Pre-treated Seaweed Residues

Variation in the ratio of enzyme buffer (50 mM sodium citrate buffer at pH 5) to pre-treated seaweed biomass (from “Investigation of Pre-treatment Conditions”) was investigated at loading rates of 2, 4, 8 and 16 % [w/v]. This was conducted in order to examine whether effective enzymatic hydrolysis could still be achieved at higher solids loading contents but still dosed with the same excess (ca. 50 FPU/g biomass) of Novozymes Cellic® CTec2, as this would make any scalable process economically attractive. Specifically it investigated whether varying the biomass to buffer ratio would have any effect on (1) the release of glucose liberated from the treated seaweed (milligrams glucose per gram seaweed), (2) the total (absolute) concentration of glucose in the enzyme liquid fraction (grams glucose per liter liquid fraction) and (3) the recovered volume of enzyme liquid fraction (mL). For process optimisation, it is important to quantify glucose in both mg g^−1^ and g L^−1^ terms, as mg g^−1^ (mg of glucose released per g of pre-treated seaweed) is indicative of enzyme hydrolysis efficacy whilst g L^−1^ indicates the actual concentration of glucose that is present in the recovered liquid fraction per liter. This is important for fermentations as high enzymatic hydrolysis efficiency (mg g^−1^) but low actual glucose concentrations (g L^−1^) would not be economical with regards to large scale fermentations [53]. The final volume (mL) of recovered liquid fractions after enzymatic hydrolysis was also important to quantify, as adequate volumes are needed in order to feasibly conduct fermentations and for accurate process efficiency calculations. For further clarification, glucose was the only carbon source that was identified in the enzyme liquid fractions post enzyme hydrolysis with Novozymes Cellic® CTec2 and no other monosaccharides were detected.

**Fig. 5:**
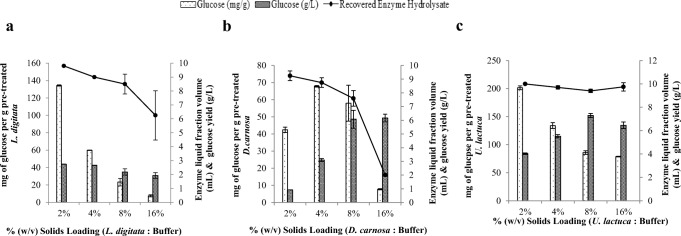
Yields of glucose (mg/g and g/L) and volumes of recovered enzyme liquid fractions (mL) from seaweeds after enzyme hydrolysis at different seaweed to enzyme buffer loading rates. **a**) *L. digitata*. **b**) *D. carnosa*. **c**) *U. lactuca*. Pre-treatment: *L. digitata* (121 °C, 1.5 N H_2_SO_4_, 24 min). *D. carnosa* and *U. lactuca* (121 °C, auto-hydrolytical, 30 min and 24 min respectively). Enzyme hydrolysis conducted at different solid loading rates (experimental volume of 10 mL) with an excess dose of Novozymes Cellic® CTec2 at 50 FPU/g biomass. Data are the mean±SD of three measurements

The enzyme solids loading rate appeared to have a direct influence on the volumes of recoverable enzyme liquid fractions and also on the amounts of glucose released per g of *L. digitata* (Fig. 5A). Yields of glucose obtained from these enzyme hydrolysis reactions were lower than the yields (253 mg g^−1^) obtained from the conditions (0.5 % (w/v) loading rate) used in preliminary experiments, which is somewhat expected (“Identification of Suitable Pre-treatment Catalyst and Conditions for Each Species of Seaweed”). For pre-treated *L. digitata*, it appeared that the lowest loading rate of 2% (w/v) generated a recoverable enzyme liquid fraction volume of 9.8 mL after hydrolysis (corresponding to 98% recovery) and contained ca 134.4 mg g^−1^ glucose. However, this corresponded to an actual glucose concentration of only 2.74 g L^−1^. This suggests that although these conditions were ideal in terms of both achieving adequate enzymatic hydrolysis efficiency (and also the recovery of an adequate volume of enzyme liquid fraction) the overall concentration of glucose (g L^−1^) may not be sufficient for economically viable bioethanol production on a commercial scale with this particular species of seaweed.

Glucose yields obtained from the enzyme hydrolysis reactions with *D. carnosa* were also lower than the yield obtained from the dilute 0.5% (w/v) loading rate used in preliminary pre-treatment screening; 229.4 mg g^−1^ (Fig. 5B). The volume of recoverable enzyme liquid fraction slightly decreased from 9.25 to 7.62 mL as the loading content increased from 2 to 8% (w/v); however the volume dropped dramatically from 7.62 to only 2.0 mL when the loading content increased from 8 to 16% (w/v). Although the highest levels of glucose in terms of mg g^−1^ were liberated from the 4% solids loading (circa 68 mg g^−1^ glucose) and the greatest concentration was yielded from the 16% solids loading rate (at just over 6 g L^−1^), the 8% solids loading rate was selected for the enzymatic hydrolysis of pre-treated *D. carnosa*. This was concluded based on the fact that it was the only solids loading rate that simultaneously yielded ‘high’ concentrations of glucose in terms of both mg g^−1^ and g L^−1^ values (57.9 mg g^−1^ and 6.1 g L^−1^, respectively), as well as generating a sufficient volume of the hydrolysate (7.62 mL). This was deemed more desirable for subsequent downstream processing of the enzyme liquid fraction into bioethanol.

In contrast to *L. digitata* and *D. carnosa*, when using *U. lactuca,* almost 10 mL of the enzyme liquid fraction was recovered. This may be because *U. lactuca* does not contain polysaccharides such as agarose, carrageenan and alginate which have strong gel forming properties, whereas ulvan (which is found predominantly in green seaweeds) forms weaker gels with lower relative viscosity in the presence of water [54]. This is an important factor to take into consideration if bioethanol is to be produced on a commercial scale. The formation of free-flowing, lower viscosity liquids is ideal in order to prevent blockages in reactors and to enable the easy transfer of the desired liquid fractions into subsequent reaction vessels. It appeared that the efficacy of the enzyme hydrolysis decreased as the solids loading content in the reactions increased; a 2% (w/v) loading content reaction yielded 201.5 mg g^−1^ glucose whereas 16% (w/v) liberated only 78.7 mg g^−1^ glucose (Fig. 5C). This did result however in a slight increase in actual glucose content in the generated enzyme liquid fraction, from 4.0 to 6.5 g L^−1^, respectively. A solids loading content of 8% (w/v) was selected as the most suitable condition for enzyme hydrolysis of *U. lactuca.* This is because the highest concentration of glucose (7.3 g L^−1^) was quantified in the 9.4 mL recovered enzyme liquid fraction. Additionally, there was only a 48.1 mg g^−1^ glucose difference between the 4 and 8% seaweed loading content reactions, which suggested that there was not a great difference in the efficacy of seaweed hydrolysis reactions between the two.

Enzyme hydrolysis investigations resulted in a decrease in the overall liberation of glucose from all three species of seaweed as the solids loading content increased. This is a known phenomenon that has been previously reported to occur and known as the ‘high-solids’ effect, where increases in solids concentration linearly decreases conversion yields [55]. A recent study identified that the high-solids effect was found to be a function of biomass-water interactions (both through water constraint and diffusion into the biomass matrix), biomass type and enzyme dependant [56]. It is important to note, however, that our present study did not represent a completely optimised process; the enzyme dosage remained the same even when the solids loading ratio increased. In order to enhance the yields of glucose that could be obtained from an optimised enzyme hydrolysis step, enzyme dosage needs to be investigated, starting with the recommended enzyme dose suggested by the supplier.

### Trial Fermentations of Seaweed Derived Enzyme Hydrolysates Using NCYC2592 *Saccharomyces cerevisiae*

**Fig. 6:**
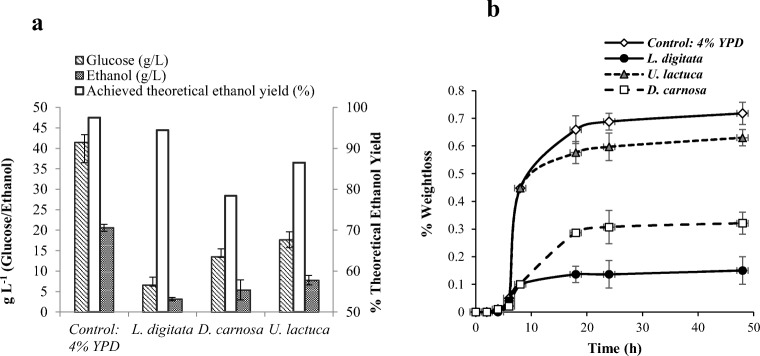
**A**) Ethanol yields and **B**) fermentation progression. Pre-treatment conditions; *L. digitata:* 1.5 N H_2_SO_4_ (121 °C 24 min) at 25% solids loading (w/v). *D. carnosa:* Auto-hydrolytical (121 °C 30 min) at 15% solids loading (w/v). *U. lactuca*: Auto-hydrolytical (121 °C 24 min) at 10% solids loading (w/v). Enzymatic saccharifications all conducted using Novozymes Cellic® CTec2 dosed at 50 FPU/g biomass at 50 °C for 48 h. Solids loading; *L. digitata:* 2% (w/v). *D. carnosa:* 8% (w/v). *U. lactuca*: 8% (w/v). Fermentations conducted at 30 °C with stirring (120 rpm) using *S. cerevisiae* NCYC2592 at a pitching rate of 1 × 10^7^ cells/mL in 25 mL of hydrolysate. Data are the mean ± SD of three replicate experiments. **A**: Theoretical ethanol yield based on based on glucose concentration in the three feedstocks. **B**: Fermentation progression monitored by weight-loss of vessels due to CO_2_ evolution

The most suitable treatment parameters for both pre-treatment and enzyme hydrolysis were applied to *L. digitata* (pre-treatment 0.75 M HCl, 121 °C, 24 min at 25 % solids loading (w/v), enzyme hydrolysis: Novozyme Cellic® CTec2 dosed at 50 FPU/g biomass, 50 °C at 2% solids loading (w/v)), *D. carnosa* (pre-treatment: Auto-hydrolytical, 121 °C, 24 min at 15% solids loading (w/v), enzyme hydrolysis: Novozyme Cellic® CTec2 dosed at 50 FPU/g biomass, 50 °C at 8% solids loading (w/v)) and *U. lactuca* (pre-treatment: Auto-hydrolytical, 121 °C, 24 min at 10% solids loading (w/v), enzyme hydrolysis: Novozyme Cellic® CTec2 dosed at 50 FPU/g biomass, 50 °C at 8% solids loading (w/v)) in order to generate enzyme liquid fractions for fermentation trials. Trial fermentations were conducted with *S. cerevisiae* NCYC2592 in order to determine which species of seaweed were able to outperform the other in terms of fermentation rate and final ethanol productivity. The achieved ethanol yields from the three seaweeds can be seen in Fig. 6A and fermentation progression profiles in Fig. 6B. Different concentrations of ethanol were produced from the three feedstocks with *L. digitata*, *D. carnosa* and *U. lactuca* producing 3.2 g L^−1^, 5.4 g L^−1^ and 7.8 g L^−1^ of ethanol, respectively. These yields equated to ca 94.5%, 78.4% and 86.5% of theoretical ethanol yield, respectively, based upon the initial content of glucose present in each of the different seaweed feedstocks and the theoretical maximum ethanol concentration that could be produced. Furthermore, despite the low bioethanol titres obtained in this study, the levels are comparable with previous studies who have acquired similar yields from *G. amansii* [8], *L. digitata* [33], *K. alvarezii* [57], *Undaria pinnatifida* [58] and *Saccharina japonica* [59].

The fermentation progression of each of the three feedstocks can be seen in Fig. 6B. All three exhibited relatively similar initial lag phase periods; however, the times of attenuation differed. It appeared that *L. digitata* feedstock reached attenuation first after ca 6 h, whereas *D. carnosa*, *U. lactuca* and the control feedstocks all achieved attenuation after ca 18 h. *Dilsea carnosa* fermentations progressed slower than the YPD control and *U. lactuca* feedstock however better than *L. digitata,* which may be possibly due to the low quantities of glucose in the feedstock. Alternatively, there may be a deficiency in assimilable nitrogen or a micronutrient (e.g. key enzyme cofactors such as zinc or magnesium) as no additional nutritional supplements were added into the feedstock hydrolysates prior to fermentation. For example, an essential trace element like zinc is crucial for the alcohol dehydrogenase enzymes that ultimately produce ethanol [60, 61] and nitrogen, in the forms of urea and ammonium sulphate, aids cell growth and viability by promoting sugar utilisation [62] and ultimately increases ethanol production [63]. In order for bioethanol production to be viable on a commercial scale, ca. 4–5% (ABV) of ethanol has to be produced [64]. As yeast convert glucose to ethanol at a maximum rate of 0.51 g ethanol (per gram of glucose), a fermentable hydrolysate containing a minimum of ca. 8% (80 g/L) glucose needs to be generated to make ethanol production from seaweed economically viable. Although the processes developed here in this work have not yet been fully optimised, the highest yield of ethanol was achieved from *U. lactuca* feedstock; reaching almost 1% (ABV).

### Bioethanol Scale up Feasibility from Seaweeds

The theoretical maximum (pure) ethanol yields were calculated based on the processing of 1 metric tonne (1000 kg; d/w) of seaweed (using the optimised pre-treatment parameters described in this study for each species of seaweed; Table 5). This provides a preliminary set of output benchmarks for any further process improvements. Regardless of seaweed species used, final ethanol yields of only ca. 15–20 kg pure ethanol per tonne of seaweed biomass could be attained. As such, these low ethanol yields would not render bioethanol production alone (e.g. without any additional bio-refining for extraction of supplementary high value products) to be economically viable, and would unlikely be able to compete with the ethanol produced from other sources (i.e. the ethanol sold today in the marketplace 1.29 US$/GAL (http://www.tradingeconomics.com/commodity/ethanol); last accessed 01/05/2019). Liberated glucose concentrations only corresponded to ca. 3–4% dry weight of the original seaweed biomass. Moreover, in addition to glucose there are other sugars such as galactose, xylose and mannitol found in seaweed, that when suitably harnessed and fermented by appropriate microorganisms (such as *Zymobacter palmae*, *Pichia angophorae* [65] and engineered strains such as *Escherichia coli* EO11 [9] and *E. coli* BAL1611 [66]) that could produce more bioethanol per tonne of seaweed. It is becoming evident however that bioethanol, or indeed any biofuel, production from seaweed biomass should be a secondary or even tertiary product derived from waste stream residues from seaweed bio-refinery processes [67–70].

**Table 5:** Summary of pre-treatment conditions for each species of seaweed, including achieved ethanol yields following fermentation and the maximal total glucose (kg) and total ethanol yields (kg) that could be attained from processing 1 metric tonne (1000 kg) of seaweed from using the optimised pre-treatment parameters

Seaweed species	Pre-treatment at 121 °C	Enzyme hydrolysis solids loading (w/v) (%)^a^	Achieved ethanol yields (g L^−1^)^b^	Total glucose (kg)^c^	Total ethanol (kg)^d^
*L. digitata*	0.75 M H_2_SO_4_, 24 min at 25% (w/v) solids loading	2	3.2	40.0	20.4
*D. carnosa*	H_2_O, 30 min at 15% (w/v) solids loading	8	5.4	30.2	15.4
*U. lactuca*	H_2_O, 24 min at 10% (w/v) solids loading	8	7.8	35.9	18.3

^a^Novozymes Cellic® CTec2 dose was not optimised in this work and as such was dosed in an excess (50 FPU/g cellulose) in order to achieve maximum glucose liberation from each seaweed

^b^After fermentation using *S. cerevisiae* NCYC2592 at 30 °C

^c^Glucose content calculated from levels quantified in the enzyme hydrolysate (hydrolysate quantity liberated from 1 tonne of seaweed)

^d^Theoretically achievable pure ethanol concentrations from 1 tonne of seaweed calculated using the liberated glucose quantity (after enzyme hydrolysis) × 0.51

Auto-hydrolytical pre-treatments are a plausible pre-treatment alternative as they are more environmentally friendly than the use of reagents such as acids. Furthermore, with the specific reaction conditions used in this work, no inhibitory sugar degradation products were formed. In order to truly decipher whether auto-hydrolytical pre-treatments are efficient for use in seaweed bioethanol processes, life cycle analysis/techno-economical assessments of the overall ‘optimised’ process would need to be performed. Only a handful of LCA studies have been performed on seaweed biorefinery systems focused on bioenergy and multiple product generation [71–74], however, by combining LCA with techno-economic, social and environmental assessments, baselines against which new biorefinery systems for added value production from seaweeds can be benchmarked for future investigation and investment [73].

## Conclusions

This study has revealed that one universal pre-treatment could not be successfully applied to different species of seaweed and pre-treatment conditions were found to be species-specific. In general, the application of a pre-treatment did generally enhance the subsequent enzymatic hydrolysis efficiencies (increased glucose yields) across the three seaweeds tested. Interestingly, results from this work showed that auto-hydrolytical treatment of the red seaweed *D. carnosa* and the green seaweed *U. lactuca* species prior to enzymatic hydrolysis enhanced glucose liberation, whereas the brown *L. digitata* required an acid thermo-chemical pre-treatment. In addition, non- pre-treated *D. carnosa* yielded similar levels of glucose to those liberated from auto-hydrolytically pre-treated residues; questioning the actual requirement of any form of pre-treatment. However, there is scope for further improvement and optimisation of the process which could ultimately enhance bioethanol production from UK species of seaweed and the results obtained from this study could be used as a starting point.
